# Aging-Associated Reductions in AMP-Activated Protein Kinase Activity and Mitochondrial Biogenesis

**DOI:** 10.1016/j.cmet.2007.01.008

**Published:** 2007-02-07

**Authors:** Richard M. Reznick, Haihong Zong, Ji Li, Katsutaro Morino, Irene K. Moore, Hannah J. Yu, Zhen-Xiang Liu, Jianying Dong, Kirsty J. Mustard, Simon A. Hawley, Douglas Befroy, Marc Pypaert, D. Grahame Hardie, Lawrence H. Young, Gerald I. Shulman

**Affiliations:** 1 Howard Hughes Medical Institute, Yale University School of Medicine, New Haven, CT 06510, USA; 2 Department of Internal Medicine, Yale University School of Medicine, New Haven, CT 06510, USA; 3 Department of Cellular & Molecular Physiology, Yale University School of Medicine, New Haven, CT 06510, USA; 4 Department of Cell Biology, Yale University School of Medicine, New Haven, CT 06510, USA; 5 Division of Molecular Physiology, University of Dundee, Dundee DD1 4HN, Scotland, UK

**Keywords:** HUMDISEASE

## Abstract

Recent studies have demonstrated a strong relationship between aging-associated reductions in mitochondrial function, dysregulated intracellular lipid metabolism, and insulin resistance. Given the important role of the AMP-activated protein kinase (AMPK) in the regulation of fat oxidation and mitochondrial biogenesis, we examined AMPK activity in young and old rats and found that acute stimulation of AMPK-α_2_ activity by 5′-aminoimidazole-4-carboxamide-1-β-D-ribofuranoside (AICAR) and exercise was blunted in skeletal muscle of old rats. Furthermore, mitochondrial biogenesis in response to chronic activation of AMPK with β-guanidinopropionic acid (β-GPA) feeding was also diminished in old rats. These results suggest that aging-associated reductions in AMPK activity may be an important contributing factor in the reduced mitochondrial function and dysregulated intracellular lipid metabolism associated with aging.

## Introduction

The AMP-activated protein kinase (AMPK) is emerging as a chief regulator of whole-body energy balance (Bolster et al., 2002, Holmes et al., 1999, Kahn et al., 2005, Kimura et al., 2003, Krause et al., 2002, Terada et al., 2002, Winder et al., 1997, Winder et al., 2000). In skeletal muscle, once AMPK becomes activated, it exerts control in part by regulating fatty-acid oxidation through the phosphorylation of acetyl-CoA carboxylase 2 (ACC2) and mitochondrial biogenesis through increasing the expression of proteins vital for proper mitochondrial function such as citrate synthase and succinate dehydrogenase (Bergeron et al., 2001, Merrill et al., 1997, Winder et al., 1997, Winder et al., 2000, Zong et al., 2002). Reductions in mitochondrial oxidative-phosphorylation activity, which contribute to dysregulated intracellular lipid metabolism, have recently been implicated in the increased incidence of insulin resistance and type 2 diabetes that occurs with aging (Petersen et al., 2003). Given the critical role that AMPK plays in the regulation of mitochondrial biogenesis and fatty-acid oxidation, we examined the effect of aging on the AMPK signaling pathway and mitochondrial biogenesis during acute pharmacological stimulation, exercise, and chronic pharmacological stimulation.

## Results and Discussion

### AICAR Infusions

**Figure 1:**
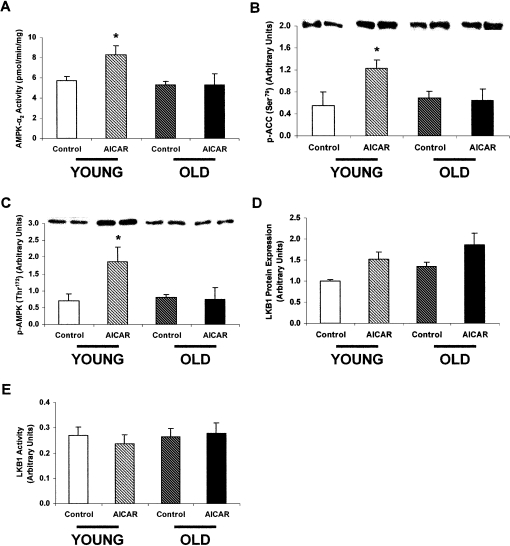
The Effect of AICAR Infusions on AMPK and LKB1 in Young and Old Rats (A) AMPK-α_2_ activity in the EDL muscle of young and old rats infused with either AICAR or saline (control). AMPK-α_2_ activity was increased by 44% in the young AICAR-treated rats compared to the young saline-treated rats. In contrast, there was no difference between the old AICAR-treated and saline-treated rats. (n = 7–10 in each group.) ^∗^p = 0.01. In this and all other figures, values are presented as means ± SEM. (B) p-ACC (Ser79) in the EDL muscle of young and old rats infused with either AICAR or saline (control). AICAR infusion resulted in a 120% increase in p-ACC (Ser79) in the young rats. In contrast, there was no effect of AICAR infusion on p-ACC (Ser79) in the old rats. (n = 4–6 in each group.) ^∗^p < 0.05. (C) p-AMPK (Thr172) in the EDL muscle of young and old rats infused with either AICAR or saline (control). AICAR infusion resulted in a 162% increase in p-AMPK (Thr172) in the young rats. In contrast, there was no effect of AICAR infusion on p-AMPK (Thr172) in the old rats. (n = 4–6 in each group.) ^∗^p < 0.05. (D) LKB1 protein expression in the EDL muscle of young and old rats infused with either AICAR or saline (control). LKB1 protein expression in EDL muscle of young and old rats did not differ significantly during aging or in response to AICAR infusion. (n = 4 in each group.) (E) LKB1 activity in the EDL muscle of young and old rats infused with either AICAR or saline (control). LKB1 activity in the EDL muscle of young and old rats did not differ significantly during aging or in response to AICAR infusion. (n = 4 in each group.)

5′-aminoimidazole-4-carboxamide-1-β-D-ribofuranoside (AICAR) is an acute activator of AMPK, which is taken up into cells and converted into 5′-aminoimidazole-4-carboxamide-1-β-D-ribofuranoside monophosphate (ZMP), which mimics the effects of AMP on AMPK (Corton et al., 1995). When bound to AMPK, AMP or ZMP induces a conformational change that makes AMPK a better substrate for activation by the upstream kinase LKB1 (Hawley et al., 2003). In vivo AICAR infusions in 3-month-old (“young”) and 28-month-old (“old”) Fisher 344 male rats increased AMPK-α_2_ activity by 44% in the extensor digitalis longus (EDL) muscles of the young AICAR-treated rats versus young saline-treated (control) rats (Figure 1A). In contrast, AICAR infusions did not increase AMPK-α_2_ activity in the old rats, demonstrating reduced AMPK activation with aging. Furthermore, AMPK phosphorylation of one of its key substrates in skeletal muscle, ACC2, at serine 79 (Ser79) was increased by 120% in the young AICAR-treated rats, whereas there was no difference in the old AICAR-treated rats (Figure 1B).

AMPK activity is regulated by the phosphorylation of threonine 172 (Thr172) on the catalytic α subunit of the enzyme (Hawley et al., 1996), and studies in tissue-specific mouse knockouts suggest that LKB1 is the major upstream kinase in skeletal muscle, at least for α_2_ (Sakamoto et al., 2005). The reduced AICAR-stimulated AMPK-α_2_ activity could be attributed to reduced phosphorylation since Thr172 phosphorylation increased by 162% in the young AICAR-treated rats and was unaltered in the old AICAR-treated rats (Figure 1C). These aging-associated differences in AMPK-α_2_ activity and Thr172 phosphorylation between AICAR-treated and saline-treated rats were independent of any changes in AMPK-α_1_ activity or in the expression of the AMPK-α_1_ and α_2_ subunits (see Figure S1 in the Supplemental Data available with this article online). They also were not associated with changes in the expression (Figure 1D) or activity of LKB1 (Figure 1E) or in the phosphorylation of Ser491 on AMPK-α_2_ (data not shown), which has been reported to antagonize phosphorylation at Thr172 (Horman et al., 2006). LKB1 occurs as long and short polypeptides (Hawley et al., 2003), which have recently been identified as splice variants (S.A.H. and D.G.H., unpublished data). Although the two forms were expressed in almost equal amounts in rat skeletal muscle by western blotting, their expression was not different in the old rats.

### Exercise

**Figure 2:**
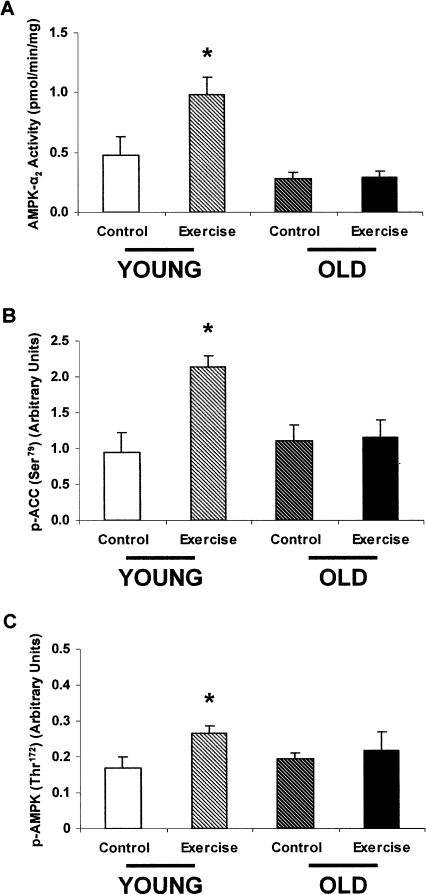
The Effect of Exercise on AMPK in Young and Old Rats (A) AMPK-α_2_ activity in the EDL muscle of exercising or sedentary young and old rats. AMPK-α_2_ activity was increased by 110% in the young exercising rats compared to the young sedentary rats. In contrast, there was no difference between the old exercising rats and old sedentary rats. (n = 8 in each group.) ^∗^p = 0.01. (B) p-ACC (Ser79) in the EDL muscle of exercising or sedentary young and old rats. Exercise resulted in a 127% increase in p-ACC (Ser79) in the young rats. In contrast, there was no effect of exercise on p-ACC (Ser79) in the old rats. (n = 4 in each group.) ^∗^p < 0.05. (C) p-AMPK (Thr172) in the EDL muscle of exercising or sedentary young and old rats. Exercise resulted in a 55% increase in p-AMPK (Thr172) in the young rats. In contrast, there was no effect of exercise on p-AMPK (Thr172) in the old rats. (n = 4 in each group.) ^∗^p < 0.05.

Exercise or contraction rapidly depletes ATP, which leads to the increase in the AMP:ATP ratio that is responsible for causing AMPK activation (Sakamoto et al., 2005, Winder and Hardie, 1996). Groups of young and old Fisher 344 male rats that had been exercise trained for 5 days completed a 5-day treadmill exercise regimen that resulted in a 110% increase in AMPK-α_2_ activity in the young rats. In contrast, the old rats displayed severely blunted AMPK-α_2_ activity in response to exercise (Figure 2A). Additionally, phosphorylation of Ser79 on ACC2 was blunted in the old rats despite a 127% increase in the young rats (Figure 2B). Lastly, Thr172 phosphorylation increased 55% in the young exercising rats and did not change in the old exercising rats compared to their controls (Figure 2C). These aging-associated differences in AMPK-α_2_ activity and Thr172 phosphorylation between exercising rats and sedentary rats were independent of any changes in AMPK-α_1_ activity or in the expression of the AMPK-α_1_ and α_2_ subunits (Figure S1).

### β-GPA Feeding

**Figure 3:**
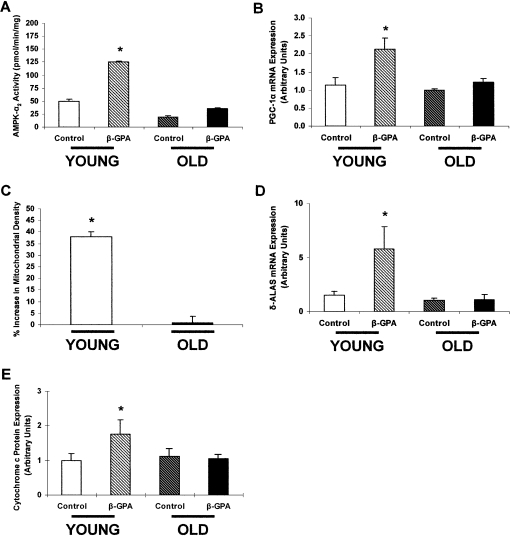
The Effect of β-GPA Feeding on AMPK and Mitochondrial Biogenesis in Young and Old Rats (A) AMPK-α_2_ activity in the EDL muscle of young and old rats fed either a β-GPA-supplemented diet or a control diet. The β-GPA-supplemented diet resulted in a 146% increase in AMPK-α_2_ activity in the young rats. In contrast, the β-GPA-supplemented diet had no effect on AMPK-α_2_ activity in the old rats. (n = 3–4 in each group.) ^∗^p < 0.01. (B) *Pgc-1*α mRNA expression in the EDL muscle of young and old rats fed either a β-GPA-supplemented diet or a control diet. *Pgc-1*α mRNA expression increased by 86% in the young β-GPA-fed rats. In contrast, there was no difference in *Pgc-1*α mRNA expression in the old rats. (n = 5–8 in each group.) ^∗^p < 0.05. (C) Percent increase in mitochondrial density in the EDL muscle of young and old rats fed a β-GPA-supplemented diet compared to control rats. Mitochondrial density increased by 38% in the young β-GPA-fed rats. In contrast, the β-GPA-supplemented diet resulted in no difference in mitochondrial density in the old rats. (n = 4–5 in each group.) ^∗^p < 0.05. (D) δ*-Alas* mRNA expression in the EDL muscle of young and old rats fed either a β-GPA-supplemented diet or a control diet. δ*-Alas* mRNA expression increased by 289% in the young β-GPA-fed rats. In contrast, there was no difference in δ*-Alas* mRNA expression in the old rats. (n = 3–8 in each group.) ^∗^p = 0.05. (E) Cytochrome *c* protein expression in the EDL muscle of young and old rats fed either a β-GPA-supplemented diet or a control diet. Cytochrome *c* protein expression increased by 76% in the young β-GPA-fed rats. In contrast, there was no difference in cytochrome *c* protein expression in the old rats. (n = 4–8 in each group.) ^∗^p < 0.05 .

Recent studies have demonstrated that AMPK serves an important role in regulating mitochondrial biogenesis during chronic energy deprivation and is associated with increased mRNA expression of peroxisome proliferator-activated receptor γ coactivator 1α (*Pgc-1*α), the transcriptional coactivator responsible for mitochondrial biogenesis in skeletal muscle. To investigate how aging impacts AMPK-stimulated mitochondrial biogenesis, we chronically fed young and old rats a diet with 1% β-guanidinopropionic acid (β-GPA) for 8 weeks. Since β-GPA has a lower K_M_ for creatine kinase than creatine, its presence results in lower intramyocellular concentrations of ATP and phosphocreatine and thus chronically stimulates AMPK activity. Accordingly, AMPK-α_2_ activity (Figure 3A) increased 146% in the EDL muscles of the young rats fed the β-GPA diet. A similar increase was detected in AMPK-α_2_ activity in the soleus tissue, an oxidative fiber, demonstrating the broad effect of β-GPA on AMPK-α_2_ activity in young rats. *Pgc-1*α mRNA expression (Figure 3B) also increased, by 86%, in the EDL muscles of the young rats fed the β-GPA diet. These increases in the young rats were associated with a 38% increase in mitochondrial density as assessed by electron microscopy (Figure 3C), a 289% increase in δ-ALA synthase (δ*-Alas*) mRNA expression (Figure 3D), and a 76% increase in cytochrome *c* protein expression (Figure 3E). In contrast, we observed no increases in AMPK-α_2_ activity, *Pgc-1*α mRNA expression, mitochondrial density, δ*-Alas* mRNA expression, or cytochrome *c* protein expression in the EDL muscles of the old rats. These results suggest that aging-associated reductions in AMPK-stimulated activity may be an important contributing factor in the reduced mitochondrial function and dysregulated intracellular lipid metabolism associated with aging-induced insulin resistance and type 2 diabetes.

## Experimental Procedures

### In Vivo AICAR Infusions

We studied Fisher 344 male rats aged 3 months (young) and 28 months (old) (National Institute on Aging). The rats were maintained on standard rat chow (Ralston Purina) and housed in an environmentally controlled room with a 12/12 hr light/dark cycle. Body weight and food consumption were measured every 2–3 days. Rats were chronically catheterized via the right jugular vein and allowed to recover (5–8 days) until they regained their preoperative weight. Weight-matched young and old animals were infused with isotonic saline (control) or the AMPK activator 5′-aminoimidazole-4-carboxamide-1-β-D-ribofuranoside (AICAR) (bolus, 100 mg/kg; constant, 10 mg/kg/min; Toronto Research Chemicals Inc.) for 60 min. During the AICAR experiments, plasma glucose concentrations were maintained constant at basal concentrations (100 mg/dl) using a variable infusion of 20% (w/v) dextrose solution in order to prevent hypoglycemia. Blood was sampled at 0 min and every 15 min for glucose measurements. At the end of the infusions, rats were anesthetized with intravenous pentobarbital (50 mg/kg), and the skeletal muscle was rapidly excised and freeze clamped in liquid nitrogen.

### Exercise

We studied Fisher 344 male rats aged 3 months (young) and 28 months (old). The rats were maintained on standard rat chow (Ralston Purina) and housed in an environmentally controlled room with a 12/12 hr light/dark cycle. Body weight and food consumption were measured every 2–3 days. The rats were introduced to the treadmill (Columbus Instruments) with a 10 min run at 10 m/min once per day for 4 days. A performance test to determine the maximum running capacity for each rat was performed on the fifth day by running the rats for 10 min at 10 m/min and then increasing the speed 1 m/min every minute until exhaustion. The young rats' maximum capacity was determined to be 38 m/min, and the old rats' maximum capacity was 20 m/min. The speed at which all of the rats reached exhaustion was noted, and the rats were run at 85% of that speed for 5 more days after a 2 day break for 30 min each day. Thus, the young rats were run at 32 m/min, and the old rats were run at 17 m/min. On the fifth day, immediately after exercise, the rats were anesthetized with isoflurane, and the skeletal muscle was rapidly excised and freeze clamped in liquid nitrogen. Throughout this experiment, control rats remained sedentary in their cages.

### β-GPA Feeding

We studied Fisher 344 male rats aged 3 months (young) and 28 months (old). The rats were maintained on standard rat chow (Ralston Purina) with or without 1% β-GPA and were housed in an environmentally controlled room with a 12/12 hr light/dark cycle. Body weight and food consumption were measured every 2–3 days. On the eighth week of feeding, the rats were chronically catheterized via the right jugular vein and allowed to recover (5–8 days) until they regained their preoperative weight. The rats were then anesthetized with intravenous pentobarbital (50 mg/kg), and the skeletal muscle was rapidly excised and freeze clamped in liquid nitrogen for analytical procedures. Other skeletal muscle was prepared separately for mitochondrial quantification using electron microscopy.

### Electron Microscopy

Individual skeletal muscle samples were prepared by immersion in 2.5% glutaraldehyde (Electron Microscopy Sciences) in 0.1 M cacodylate buffer (pH 7.4) at 4°C overnight. Skeletal muscles were then washed three times in 0.1 M cacodylate buffer (pH 7.4) and postfixed for 1 hr in 1% osmium tetroxide (EMbed-812 epoxy resin) in the same buffer at room temperature. After three washes in water, the samples were stained for 1 hr at room temperature in 2% uranyl acetate, washed again in water, and dehydrated in a graded series of ethanol dilutions (50%–100%). The samples were then embedded in EMbed. Ultrathin (60 nm) sections were cut using a Reichert Ultracut ultramicrotome, collected on formvar- and carbon-coated grids, stained with 2% uranyl acetate and lead citrate, and examined in a Philips 410 electron microscope. Only cross-sections of skeletal muscle were examined for quantification of mitochondrial density. For each individual rat and skeletal muscle, six random pictures were taken at a magnification of 7,100× and printed at a final magnification of 18,250×. The volume density of mitochondria was estimated using the point-counting method. The average volume density was calculated for each individual rat and skeletal muscle and was used to calculate the average volume density for each treatment.

### AMPK Activity Assays

At the end of the experiments, the rats were anesthetized as described above. The EDL muscles from the left and right legs were quickly freeze clamped in situ. Skeletal muscle samples were kept in liquid nitrogen until analyzed. EDL was ground with a mortar and pestle and mixed with 1 ml of lysis buffer (50 mM Tris-HCl buffer [pH 7.5 at 4°C], 50 mM NaF, 5 mM NaPPi, 1 mM EDTA, 1 mM EGTA, 1 mM dithiothreitol [DTT], 1 mM benzamidine, 1 mM phenylmethanesulfonyl fluoride [PMSF], glycerol [10% v/v], Triton X-100 [1% v/v]). Homogenates were spun at 20,800 × g for 10 min at 4°C, and protein concentrations were determined. AMPK-α_1_ and AMPK-α_2_ were immunoprecipitated overnight from cell lysates containing 1 mg of protein using 1 μl of either AMPK-α_1_ antibody or AMPK-α_2_ antibody (Santa Cruz Biotechnology). Skeletal muscle AMPK-α_1_ and AMPK-α_2_ activity were determined by following the incorporation of [^32^P]ATP into a synthetic peptide containing the AMARA sequence on the following day.

### LKB1 Activity Assays

EDL muscle samples were frozen in liquid nitrogen and later ground with a mortar and pestle. For each sample, the ground paste was extracted in 1 ml of lysis buffer as described above. Homogenates were spun at 12,900 rpm for 10 min at 4°C, and protein concentration was estimated. LKB1 was immunoprecipitated from lysates containing 3 mg of protein using the N-terminal antipeptide antibody (Boudeau et al., 2003). LKB1 assays (1 mg of lysate per assay) were conducted using purified GST-AMPK-α_1_ catalytic subunit as substrate in shaking incubators as described previously for immunoprecipitate kinase assays of AMPK (Hardie et al., 2000).

### Real-Time Quantitative PCR Analysis

Total RNA was isolated from the EDL muscle using the RNeasy Kit (QIAGEN). cDNA was prepared from 2 μg of RNA using the StrataScript RT-PCR kit (Strategene) with random hexamer primers according to the manufacturer's instructions. The resulting cDNA was diluted, and a 4 ng aliquot was used in a 40 μl PCR reaction using SYBR green and selected primers. The primers for δ*-Alas* were 5′-TTTGTGGACGAGGTCCATGCAGTA-3′ (forward) and 5′-GCATTCAGCTGACGAATGTGGCTT-3′ (reverse). The primers for cytochrome *c* were 5′-TCTTGACTTCCTGACCTTGGGCTT-3′ (forward) and 5′-ACTCCCGCTTCTTGTAGCTTTCCA-3′ (reverse). PCR reactions were run in duplicate and quantitated with an ABI Prism 7700 Sequence Detection System (Applied Biosystems). Cycle threshold values were normalized to β*-actin* mRNA expression.

### Western Blotting

For all western blots except for LKB1 expression, 30 μg of cell lysate was diluted in LDS Laemmli sample buffer before SDS-PAGE. SDS gel electrophoresis was performed using precast Bis-Tris 4%–12% gradient polyacrylamide gels in the MOPS buffer system (Invitrogen). After transfer to nitrocellulose membranes, membranes were incubated in blocking buffer (5% milk) for 1 hr and immunoblotted with anti-p-AMPK (Thr172) antibody (Cell Signaling), anti-p-ACC (Ser79) antibody (Cell Signaling), anti-AMPK-α_1_ antibody (Cell Signaling), anti-AMPK-α_2_ antibody (Cell Signaling), or cytochrome *c* antibody (Santa Cruz Biotechnology) at 1:1000 dilution in 5% bovine serum albumin. The membranes were washed three times for 15 min with TBS (10 mM Tris-HCl [pH 7.4], 0.5 M NaCl) plus Tween 20 (0.2% v/v) (TBST). The membranes were immersed in blocking buffer and a corresponding IgG-conjugated secondary antibody and were shaken for 1 hr. The membranes were then washed three times for 5 min using TBST. Proteins were then detected with enhanced chemiluminescence, and autoradiographs were quantified using densitometry. For western blotting of LKB1, 15 μg of tissue extract was diluted in LDS sample buffer (Invitrogen). SDS gel electrophoresis was performed using precast Bis-Tris 4%–12% gradient polyacrylamide gels in the MOPS buffer system (Invitrogen). Proteins were transferred to nitrocellulose membranes (Bio-Rad) using the Xcell II Blot Module (Invitrogen). Membranes were incubated in Odyssey blocking buffer (Li-Cor) for 1 hr. The membrane was subsequently incubated with 1:1000 anti-LKB1 N-terminal antibody (raised against residues 24–36), which had been diluted in Odyssey blocking buffer containing 0.2% Tween 20. The membranes were washed six times for 5 min with TBST. The membranes were immersed in blocking buffer containing Tween 20 (0.2% v/v) and 1 μg/ml anti-sheep IgG conjugated to IR dye 680 (Molecular Probes) and shaken for 1 hr, protected from light. The membranes were then washed six times for 5 min using TBST and once for 5 min in PBS. The membranes were scanned in the 700 nm channel using the Odyssey IR imager, and LKB1 protein levels were quantified using Odyssey IR imaging system software.

### Statistical Analyses

All data are reported as means ± SEM. Results were analyzed using Student's t test. Differences were considered statistically significant at p < 0.05.

## Supplemental Data

Document S1. One Figure
