# Supplementary material for: Aging-Associated Reductions in AMP-Activated Protein Kinase Activity and Mitochondrial Biogenesis
Source: Cell Metab. 2007 Feb 7;5(2):151–6. doi: 10.1016/j.cmet.2007.01.008 (PMC1885964; doi:10.1016/j.cmet.2007.01.008)
Supplement: Document S1. One Figure [file mmc1.pdf]

Cell Metabolism, Volume *5*

Supplemental Data

Short Article

Aging-Associated Reductions in AMP-Activated Protein Kinase Activity and Mitochondrial Biogenesis

Richard M. Reznick, Haihong Zong, Ji Li, Katsutaro Morino, Irene K. Moore, Hannah J. Yu, Zhen-Xiang Liu, Jianying Dong, Kirsty J. Mustard, Simon A. Hawley, Douglas Befroy, Marc Pypaert, D. Grahame Hardie, Lawrence H. Young, and Gerald I. Shulman

Figure S1.

(A) AMPK-α_1 activity in the EDL muscle of young and old rats infused with either AICAR or saline (Control). AMPK-α_1 activity was not increased in the young AICAR-treated rats compared to the young saline-treated rats. Additionally, there was no difference between the old AICAR-infused and saline-infused rats. All values are means ± SE. (n = 4 in each group.)

(B) AMPK-α_1 protein expression in the EDL muscle of young and old rats infused with either AICAR or saline (Control). AMPK-α_1 protein expression was not increased in the young AICAR-treated rats compared to the young saline-treated rats. Additionally, there was no difference between the old AICAR-infused and saline-infused rats. All values are means ± SE. (n = 4 in each group.)

(C) AMPK-α_2 protein expression in the EDL muscle of young and old rats infused with either AICAR or saline (Control). AMPK-α_2 protein expression was not increased in the young AICAR-treated rats compared to the young saline-treated rats. Additionally, there was no difference between the old AICAR-infused and saline-infused rats. All values are means ± SE. (n = 4 in each group.)

(D) AMPK-α_1 activity in the EDL muscle of exercising and sedentary (Control) young and old rats. AMPK-α_1 activity was not increased in the young exercising rats compared to the young sedentary rats. Additionally, there was no difference between the old exercising and sedentary rats. All values are means ± SE. (n = 4 in each group.)

(E) AMPK-α_1 protein expression in the EDL muscle of exercising and sedentary (Control) young and old rats. AMPK-α_1 protein expression was not increased in the young exercising rats compared to the young sedentary rats. Additionally, there was no difference between the old exercising and sedentary rats. All values are means ± SE. (n = 4 in each group.)

(F) AMPK-α_2 protein expression in the EDL muscle of exercising and sedentary (Control) young and old rats. AMPK-α_2 protein expression was not increased in the young exercising rats compared to the young sedentary rats. Additionally, there was no difference between the old exercising and sedentary rats. All values are means ± SE. (n = 4 in each group.)

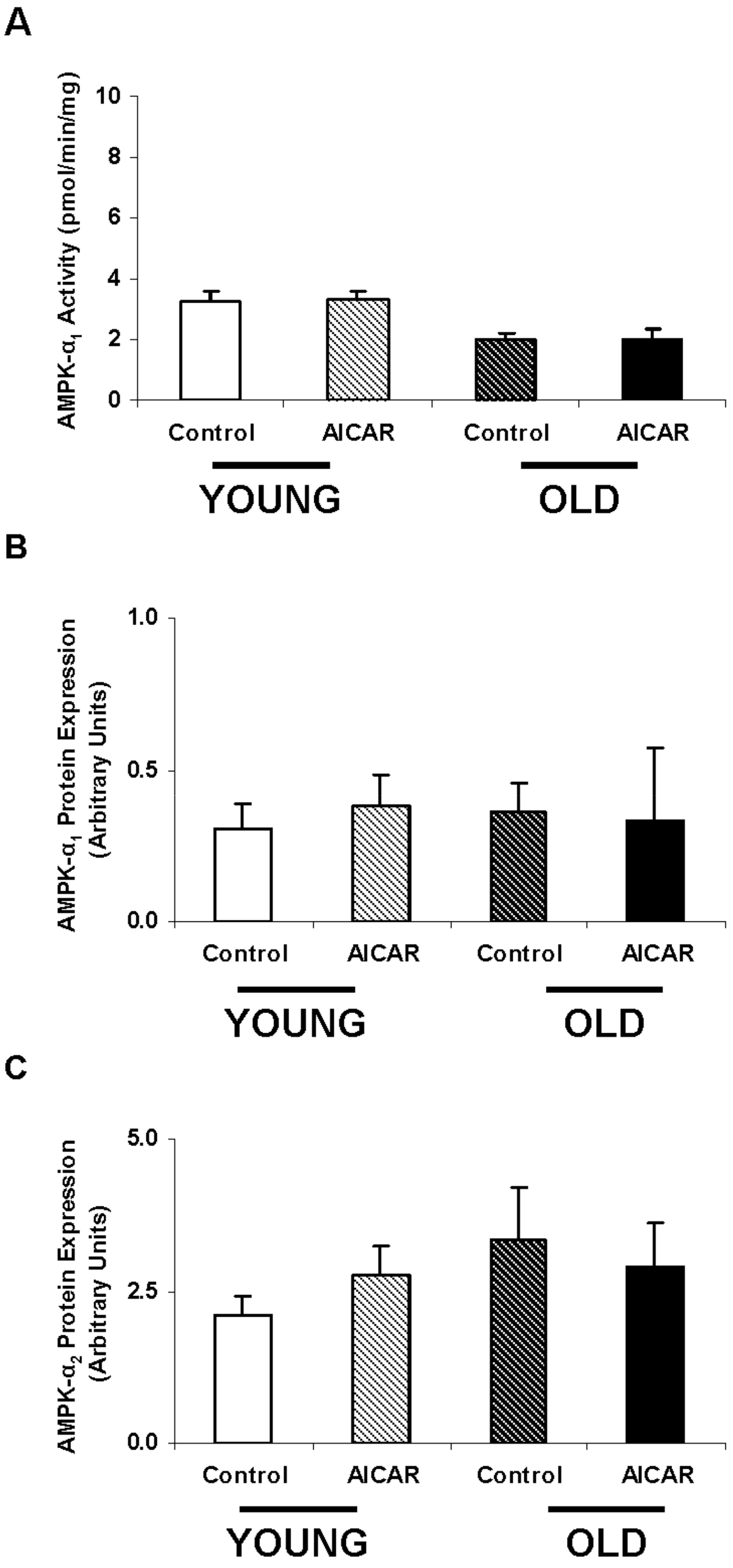
A
AMPK-α1 Activity (pmol/min/mg)
10
8
6
4
2
0
Control
AICAR
Control
AICAR
YOUNG
OLD
B
AMPK-α1 Protein Expression (Arbitrary Units)
1.0
0.5
0.0
Control
AICAR
Control
AICAR
YOUNG
OLD
C
AMPK-α2 Protein Expression (Arbitrary Units)
5.0
2.5
0.0
Control
AICAR
Control
AICAR
YOUNG
OLD

D

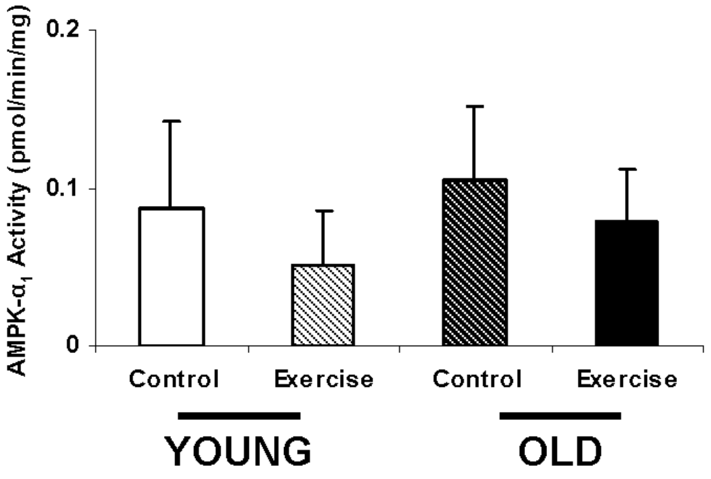
AMPK-α_1 Activity (pmol/min/mg)
0.2
0.1
0
Control
Exercise
Control
Exercise
YOUNG
OLD

E

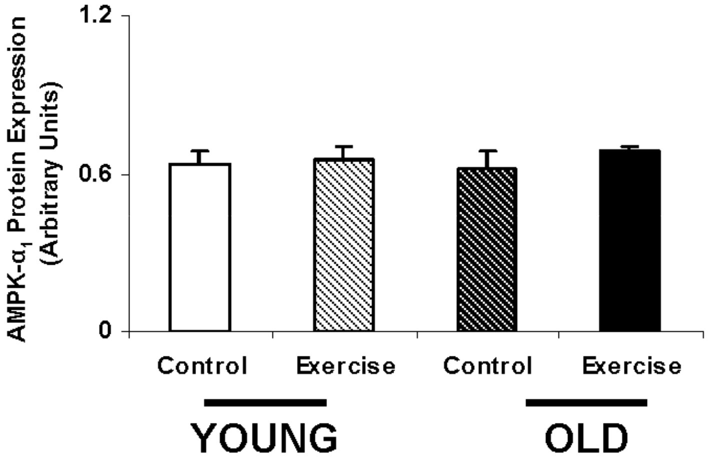
AMPK-α_1 Protein Expression (Arbitrary Units)
1.2
0.6
0
Control
Exercise
Control
Exercise
YOUNG
OLD

F

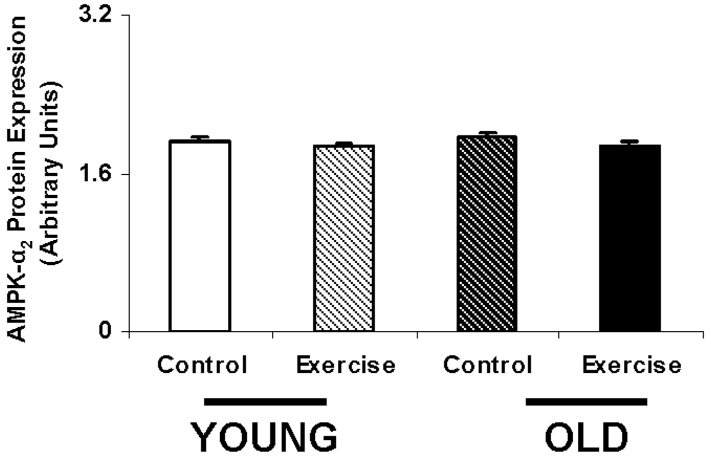
AMPK-α_2 Protein Expression (Arbitrary Units)
3.2
1.6
0
Control
Exercise
Control
Exercise
YOUNG
OLD
